# Endoscopic Resection Versus Surgery in the Treatment of Early Gastric Cancer: A Systematic Review and Meta-Analysis

**DOI:** 10.3389/fonc.2022.939244

**Published:** 2022-07-12

**Authors:** Alexandre Moraes Bestetti, Diogo Turiani Hourneaux de Moura, Igor Mendonça Proença, Epifanio Silvino do Monte Junior, Igor Braga Ribeiro, João Guilherme Ribeiro Jordão Sasso, Angelo So Taa Kum, Sergio A. Sánchez-Luna, Wanderley Marques Bernardo, Eduardo Guimarães Hourneaux de Moura

**Affiliations:** ^1^Serviço de Endoscopia Gastrointestinal do Hospital das Clínicas Hospital das Clínicas da Faculdade de Medicina da Universidade de São Paulo (HCFMUSP), Departamento de Gastroenterologia, Faculdade de Medicina, Universidade de Sao Paulo, Sao Paulo, SP, Brazil; ^2^Basil I. Hirschowitz Endoscopic Center of Excellence, Division of Gastroenterology & Hepatology, Department of Internal Medicine, The University of Alabama at Birmingham Heersink School of Medicine, Birmingham, AL, United States

**Keywords:** gastric cancer, early gastric cancer, ESD, EMR, gastrectomy, endoscopy

## Abstract

**Background and Aim:**

Endoscopic resection (ER) is the preferred approach to treat early gastric cancer (EGC) in patients without suspected lymph node involvement and that meet the criteria for ER. Surgery is a more aggressive treatment, but it may be associated with less recurrence and the need for reintervention. Previous meta-analyses comparing ER with surgery for EGC did not incorporate the most recent studies, making accurate conclusions not possible.

**Methods:**

This systematic review and meta-analysis aimed to examine complete resection, length of hospital stay (LOHS), adverse events (AEs), serious AEs, recurrence, 5-year overall survival (OS), and 5-year cancer-specific survival (CSS) in patients with EGC.

**Results:**

A total of 29 cohorts studies involving 20559 patients were included. The ER (n = 7709) group was associated with a lower incidence of AEs (RD = -0.07, 95%CI = -0.1, -0.04, p < 0.0001) and shorter LOHS (95% CI -5.89, -5.32; p < 0,00001) compared to surgery (n = 12850). However, ER was associated with lower complete resection rates (RD = -0.1, 95%CI = -0.15, -0.06; p < 0.00001) and higher rates of recurrence (RD = 0.07, 95%CI = 0.06; p < 0.00001). There were no significant differences between surgery and ER in 5-year OS (RD = -0.01, 95%CI = -0.04, 0.02; p = 0.38), 5-year CSS (RD = 0.01, 95%CI = 0.00, 0.02; p < 0.17), and incidence of serious AEs (RD = -0.03, 95%CI = -0.08, 0.01; p = 0.13).

**Conclusions:**

ER and surgery are safe and effective treatments for EGC. ER provides lower rates of AEs and shorter LOHS compared to surgery. Although ER is associated with lower complete resection rates and a higher risk of recurrence, the OS and CSS were similar between both approaches.

**Systematic Review Registration:**

https://www.crd.york.ac.uk/PROSPERO/, identifier CRD42021255328.

## Introduction

Gastric cancer is one of the most common cancers worldwide, being the main cause of death by cancer in the world until the mid-1980s. In the last decades, there has been a substantial decline in its incidence, fundamentally due to recognizing and controlling of risk factors, such as diet, smoking, and *Helicobacter pylori* infection (1). Despite this, gastric adenocarcinoma is still the fifth most common neoplasm in the world (2), with a poor prognosis due to a generally late diagnosis.

Early gastric cancer (EGC) has been defined by the Japanese Society of Endoscopy as adenocarcinoma involving mucosa or submucosa, regardless of lymph node status (3). Despite achieving good oncological results, with wide resection margins and lymphadenectomy, surgical treatment has been typically associated with significant morbidity and potential impact on patients’ quality of life (4). Thus, the development of advanced endoscopic resection (ER) techniques such as endoscopic mucosectomy resection (EMR) or endoscopic submucosal dissection (ESD) has enabled less invasive treatment for patients at low risk of lymph node metastasis (5).

The absolute criteria for indication of ER in EGC are well or moderately-differentiated intramucosal adenocarcinoma up to 2 centimeters (cms) with no associated ulceration. However, with several improvements related to the ER techniques, the resection criteria have become obsolete, leading to unnecessary surgical indications (6). Thus, based on encouraging studies from Asian centers, expanded criteria for endoscopic resection was proposed by Gotoda et al. in 2007, including 1) intramucosal cancer, differentiated, without ulceration, regardless of size; 2) intramucosal cancer, differentiated, with ulceration, and < 3 cms in diameter; 3) intramucosal cancer, undifferentiated histology, not ulcerated, and smaller than 2 cms in diameter; and 4) differentiated cancer < 3 cms, not ulcerated, and with submucosal invasion <0.5 mm (7).

To date, there is no randomized clinical trial (RCT) comparing endoscopic versus surgical treatment for EGC and previous metanalyses did not include several recent relevant studies. Due to the important evolution in resection techniques with the emergence of new devices, higher quality equipment, increase in trained professionals for the procedure, and increase in the number of resections worldwide, including the western countries (8), several observational studies have addressed the topic over the last few years. Therefore, an updated meta-analysis is warranted for an adequate understanding of the current status of EGC treatment.

## Materials and Methods

### Protocol and Registration

This systematic review and meta-analysis was performed in conformity with the recommendations from the Cochrane Handbook of Systematic Reviews of Interventions and the Preferred Reporting Items for Systematic Reviews and Meta-analysis (PRISMA) guidelines (9). The study protocol was registered in the International Prospective Register of Systematic Reviews (PROSPERO) under the file number CRD42021255328 and was approved by the Ethics Committee of Hospital das Cliínicas, Faculty of Medicine at the University of São Paulo.

### Eligibility Criteria

We screened clinical trials and comparative observational studies of adequate quality, comparing endoscopic versus surgical treatment in patients with EGC. No restrictions were made for publication date. The exclusion criteria were non-comparative studies and studies published in a language other than English. When articles with a concern of sample duplication were identified, only the most recent was included. Studies with missing data and failed contact attempts were also excluded.

### Search Strategy and Information Sources

Studies were identified by searching electronic databases (MEDLINE, EMBASE, Lilacs, and Central Cochrane) and grey literature from inception through January 11th, 2022. The search strategy for MEDLINE was: [(Stomach Neoplasms OR Stomach Neoplasm OR Gastric Neoplasms OR Gastric Neoplasm OR Cancer of Stomach OR Stomach Cancers OR Gastric Cancer OR Gastric Cancers OR Stomach Cancer) AND (Endoscopic OR Endoscopy) AND (Surgery OR Surgical OR Operative)]. We used the same or equivalent strategy for searching in the remaining databases.

### Study Selection and Data Collection Process

Two independent authors accessed all records in the aforementioned sources by titles. Potentially relevant studies were screened for eligibility by abstracts. If an abstract matched the eligibility criteria, or if it was unclear, the full text was accessed. Duplicates were removed. Any differences were resolved by mutual agreement and consultation with a third reviewer. The researchers used Excel spreadsheets to extract the data and relevant results.

### Data Items

After the selection for final analysis, the information was extracted based on: (1) characteristics of study participants (age and pattern of different types of EGC), inclusion and exclusion criteria, length of follow-up; (2) interventions (considering different modalities in the endoscopic treatment and surgical approaches); and (3) outcomes (adverse events (AEs), serious AEs (AEs), length of hospital stay (LOHS), survival rates, mortality, recurrence, and complete resection rates).

Complete resection was defined as margins free of neoplastic or high-grade intraepithelial dysplasia after a surgical or endoscopic procedure.

LOHS considers the whole hospital internment, from admission for the proposed procedure until hospital discharge. AE include any procedural-related event and were evaluated based on the Clavien Dindo Score. Serious AEs were defined as a Clavien Dindo ≥ 3 (10).

Recurrence was characterized by the reappearance of gastric cancer after treatment. Both local and distant recurrence was considered in our analysis.

Five-year overall survival refers to the percentage of people who are still alive 5 years after the treatment was performed. Otherwise, 5-year cancer-specific survival considers only cancer-related deaths.

### Risk of Bias and Quality of Evidence

The risk of bias was assessed by Cochrane’s Risk of Bias in Non-randomized Studies of Interventions (ROBINS-I) (9) and the quality of evidence was assessed using the objective criteria of Grading of Recommendations Assessment, Development, and Evaluation (GRADE) for each outcome using the GRADEpro - Guideline Development Tool software (11).

### Risk of Bias and Quality of Evidence

The data from the selected studies were meta-analyzed through the software Review Manager version 5.4 (RevMan 5.4). For dichotomous endpoints, the difference was calculated by the risk difference (RD), using the Cochrane Mantel-Haenszel test, with 95% confidence interval (CI). Heterogeneity (inconsistency) was assessed and quantified according to the chi-square (χ2) and Higgins method (I2). Heterogeneity (I2) values greater than 50% were considered high, with a random-effects model chosen to evaluate this data given associated heterogeneity in the meta-analysis. For heterogeneity values less than 50%, a fixed-effects model was employed. P < 0.05 was considered statistically significant. For continuous variables, the inverse variance test was applied. To calculate the differences between the measures, the mean difference was used through calculations among the mean, standard deviation, and sample size of each group. In the studies where the standard deviation was not reported, it was calculated using the mean, the interval reported in the outcome, and the sample size.

## Results

### Overview

**Figure 1:**
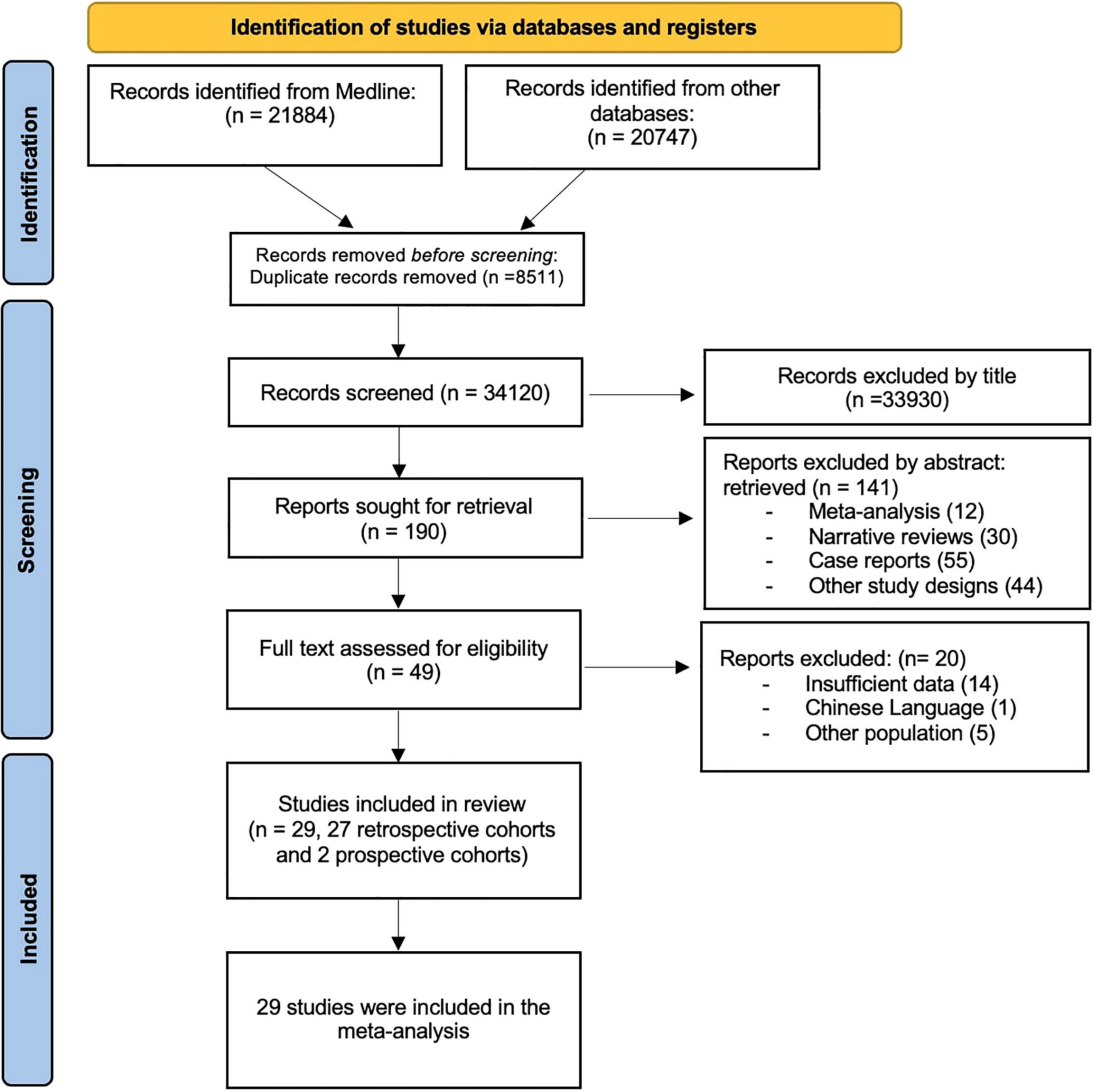
PRISMA flow diagram.

After an initial search, 34210 studies were evaluated. After excluding duplicate studies and applying the inclusion and exclusion criteria, 29 studies (12–40) were included for quantitative and qualitative analysis (**Figure 1**).

### Characteristics of the Studies

The 29 studies selected were cohorts (2 prospective and 27 retrospective) of patients who underwent endoscopic (ER) or surgical (SG) treatment of EGC, 25 of which included patients with expanded indication (EI) while 4 only patients with absolute indication (AI) for endoscopic resection. The included records involved 20559 patients (7709 in the ER group and 12850 in the SG group) (**Table 1**).

**Table 1:** Characteristics of the studies.

Author	Study design	Patients	Inclusion criteria	Outcomes
Kamarajah et al [33452601], 2021	RC	5842 (EG = 1631; SG = 4211)	AI + EI	5yr OS, CR
Ahn et al [33211219], 2020	RC	436 (EG = 218; SG = 218)	AI + EI	5yr OS, Recurrence
Yang et al [31876838], 2020	RC	474 (EG = 176; SG = 298)	AI + EI	5yr OS, 5yr CSS
Hong et al [32900577], 2020	RC	137 (EG = 36; SG = 101)	AI + EI	Hospital stay, AE, CR, Recurrence
Quero et al [32483697], 2020	RC	84 (EG = 42; SG = 42)	AI	5yr OS, Hospital stay, AE, SAE, CR
Guo et al [32481468], 2020	RC	92 (EG = 40; SG = 52)	AI + EI	5yr OS, AE, Recurrence
Pourmousavi et al [32389885], 2020	RC	3363 (EG = 786; SG = 2577)	AI	5yr OS, 5yr CSS
Zhao et al [31983126], 2019	RC	194 (EG = 58; SG = 136)	AI + EI	Hospital stay, AE, CR, Recurrence
Lim et al [30604260], 2019	RC	474 (EG = 102; SG = 372)	AI + EI	5yr OS, CR, Recurrence, 5yr CSS
Bausys et al [30511310], 2018	RC	84 (EG = 42; SG = 42)	AI + EI	5yr OS, Hospital stay, AE, SAE, CR, Recurrence
Libânio et al [29969807], 2018	PC	254 (EG = 153; SG = 101)	AI + EI	Hospital stay, AE, SAE, CR
Kim et al [29067581], 2018	PC	161 (EG = 48; SG = 113)	AI	5yr OS, AE, SAE
Park et al [29052072], 2018	RC	162 (EG = 81; SG = 81)	AI + EI	Hospital stay, AE, CR, Recurrence
Lee et al [29052052], 2018	RC	1044 (EG = 522; SG = 522)	AI + EI	5yr OS, Hospital stay, AE, SAE, Recurrence, 5yr CSS
Hahn et al [28639042], 2018	RC	2023 (EG = 817; SG = 1206)	AI + EI	5yr OS, AE, Recurrence, 5yr CSS
Chang et al [28746176], 2017	RC	153 (EG = 74; SG = 79)	AI + EI	5yr OS, AE, SAE, CR, Recurrence, 5yr CSS
Jeon et al [28397011], 2017	RC	617 (EG = 342; SG = 275)	AI + EI	5yr OS, Hospital stay, AE, 5yr CSS
Fukunaga et al [27365265], 2017	RC	148 (EG = 74; SG = 74)	AI + EI	5yr OS, AE
Ryu et al [27338583], 2016	RC	225 (EG = 81; SG = 144)	AI + EI	5yr OS, Hospital stay, AE, CR, Recurrence
Najmeh et al [27282756], 2016	RC	67 (EG = 30; SG = 37)	AI + EI	Hospital stay, AE, SAE, CR
Shin et al [27157856], 2016	RC	275 (EG = 175; SG = 100)	AI + EI	5yr OS, Hospital stay, AE, CR, Recurrence
Pyo et al [26782817], 2016	RC	2563 (EG = 1290; SG = 1273)	AI + EI	Hospital stay, AE, CR, Recurrence
Cho et al [26659226], 2016	RC	176 (EG = 88; SG = 88)	AI + EI	AE, Recurrence
Song et al [26537433], 2015	RC	88 (EG = 29; SG = 59)	AI + EI	Hospital stay, AE, CR
Kim et al [25625697], 2015	RC	457 (EG = 165; SG = 292)	AI + EI	5yr OS, AE, SAE, Recurrence, 5yr CSS
Choi et al [25281498], 2015	RC	375 (EG = 261; SG = 114)	AI	5yr OS, AE, Recurrence
Park et al [24973177], 2014	RC	264 (EG = 132; SG = 132)	AI + EI	5yr OS, Hospital stay, AE, Recurrence
Kim et al [25228976], 2014	RC	213 (EG = 142; SG = 71)	AI + EI	Hospital stay, AE, Recurrence
Chiu et al [22678176], 2012	RC	114 (EG = 74; SG = 40)	AI + EI	Hospital stay, AE

RC, Retrospective cohort; PC, Prospective cohort; EG, Endoscopic Group; SG, Surgical Group; AI, Absolute Indications; EI, Expanded Indications; OS, Overall Survival; AE, Adverse Events; SAE, Severe Adverse Events; CR, Complete Resection; CSS, Cancer-Specific Survival.

### Risk of Bias and Quality of the Evidence

The risk of bias was moderate for all included studies assessed by Robins-I (**Table 2**). The quality of evidence evaluated by the GRADE for 5-year overall survival, LOS, AEs, complete resection, and 5-year cancer-specific survival were low; for serious AEs was moderate, and for recurrence was high (**Table 3**).

**Table 2:** Risk of bias assessment assessed by ROBINS-I.

	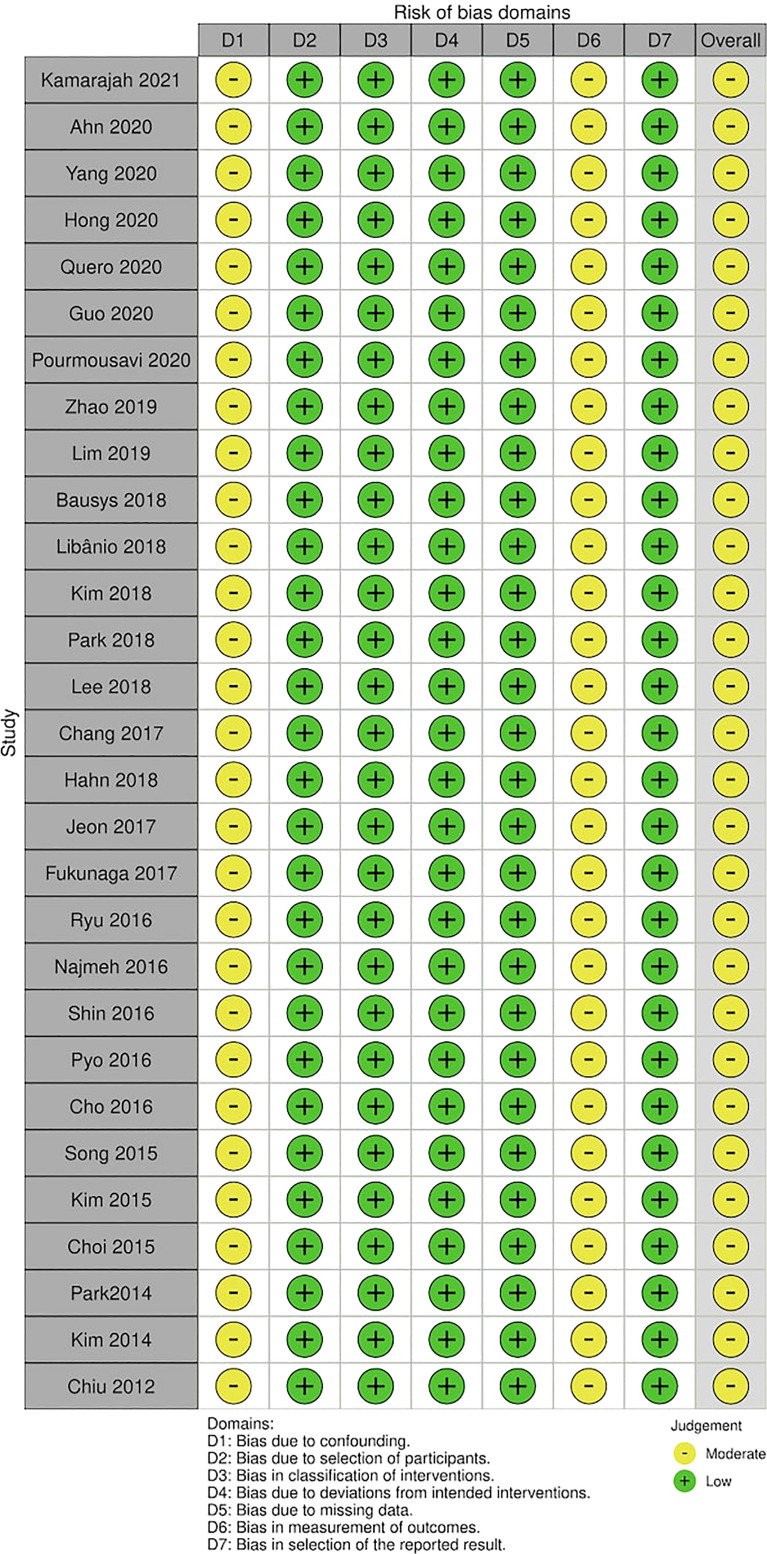

**Table 3:** Quality of evidence evaluated by GRADE.

Certainty assessment							Summary of findings				
Participants (studies) Follow-up	Risk of bias	Inconsistency	Indirectness	Imprecision	Publication bias	Overall certainty of evidence	Study event rates (%)		Relative effect (95% CI)	Anticipated absolute effects	
							With Surgery	With Endoscopic Ressection		Risk with Surgery	Risk difference with Endoscopic Ressection
**5y overall survival**											
16591 (19 obs ervational s tudies )	seriousa	very seriousb	not serious	not serious	all plausible residual confounding would reduce the demonstrated effect	⨁⨁◯◯ Low	8896/10863 (81.9%)	4688/5728 (81.8%)	**RR 0.98** (0.95 to 1.02)	819 per 1.000	**16 fewer per 1.000** (from 41 fewer to 16 more)
**Lenght of hospital stay**											
6385 (16 obs ervational s tudies )	seriousa	very seriousb	not serious	not serious	all plausible residual confounding would reduce the demonstrated effect	⨁⨁◯◯ Low	3156	3229	–	The mean lenght of hospital stay was **0**	MD **5.61 lower** (5.89 lower to 5.32 lower)
**Adverse Events (total)**											
9960 (24 obs ervational s tudies )	seriousa	very seriousb	not serious	not serious	all plausible residual confounding would reduce the demonstrated effect	⨁⨁◯◯ Low	750/5174 (14.5%)	385/4786 (8.0%)	**RR 0.55** (0.43 to 0.71)	145 per 1.000	**65 fewer per 1.000** (from 83 fewer to 42 fewer)
**Serious Adverse Events**											
2304 (8 obs ervational s tudies )	seriousa	seriousc	not serious	not serious	all plausible residual confounding would reduce the demonstrated effect	⨁⨁⨁◯ Moderate	81/1228 (6.6%)	53/1076 (4.9%)	**RR 0.75** (0.42 to 1.33)	66 per 1.000	**16 fewer per 1.000** (from 38 fewer to 22 more)
**Complete Resection**											
10602 (14 obs ervational s tudies )	seriousa	very seriousb	not serious	not serious	all plausible residual confounding would reduce the demonstrated effect	⨁⨁◯◯ Low	6656/6778 (98.2%)	3269/3824 (85.5%)	**RR 0.90** (0.85 to 0.95)	982 per 1.000	**98 fewer per 1.000** (from 147 fewer to 49 fewer)
**Recurrence**											
9245 (18 obs ervational s tudies )	seriousa	not serious	not serious	not serious	very strong as s ociation all plausible residual confounding would reduce the demonstrated effect	⨁⨁⨁⨁ High	55/4983 (1.1%)	330/4262 (7.7%)	**RR 6.76** (5.10 to 8.97)	11 per 1.000	**64 more per 1.000** (from 45 more to 88 more)
**5-year cancer specific survival**											
8605 (8 obs ervational s tudies )	seriousa	very seriousb	not serious	not serious	all plausible residual confounding would reduce the demonstrated effect	⨁⨁◯◯ Low	5434/5621 (96.7%)	2946/2984 (98.7%)	**RR 1.01** (1.00 to 1.02)	967 per 1.000	**10 more per 1.000** (from 0 fewer to 19 more)

**CI**, confidence interval; **MD**, mean difference; **RR** risk ratio.

**Explanations**

a. Observational studies.

b. Very high heterogeneity.

c. High heterogeneity.

### Meta-Analysis

#### Complete Resection

**Figure 2:**
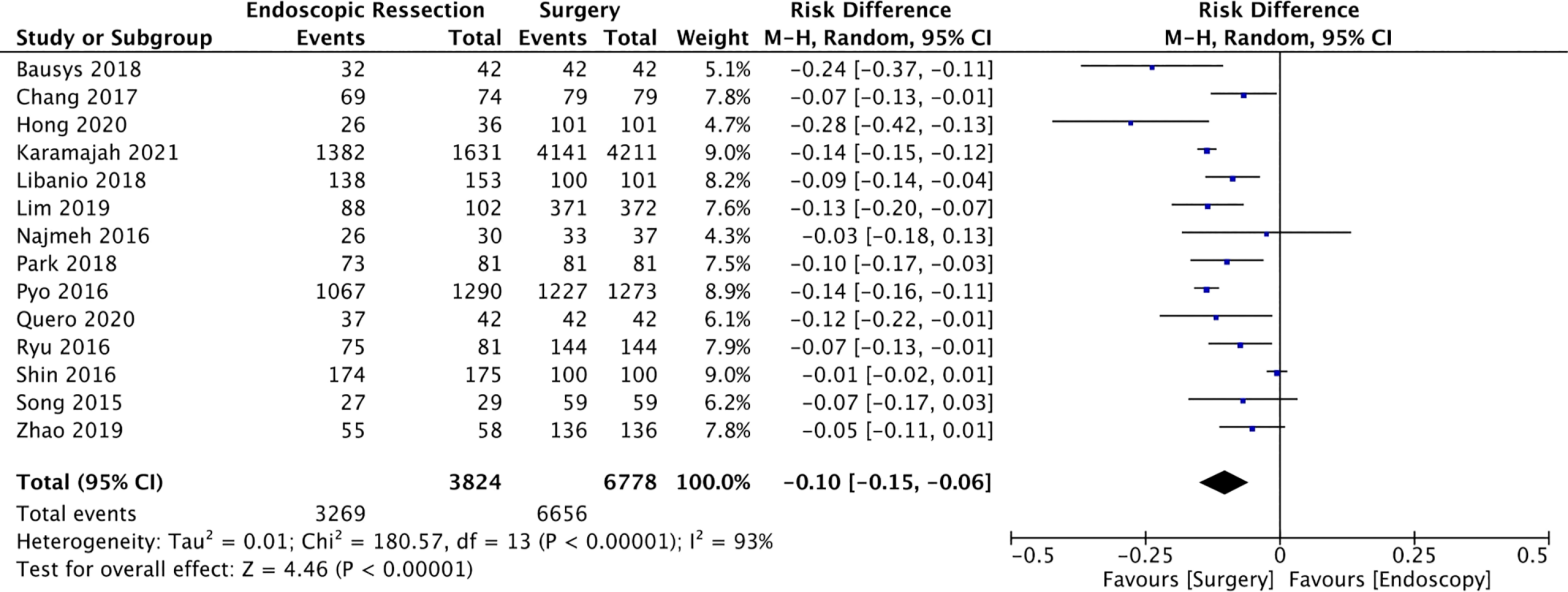
Forest Plot Complete Resection.

Fourteen studies (12, 14, 16, 19, 22, 23, 25, 26, 28, 34, 35, 38–40) were included in this analysis, totaling 10602 patients (3824 in the ER group and 6778 in the SG group). Complete resection rate was higher in the SG group (RD = -0.1, 95%CI = -0.15, -0.06; I² = 93%; *p* < 0.00001) (**Figure 2**).

#### Length of Hospital Stay (LOHS)

**Figure 3:**
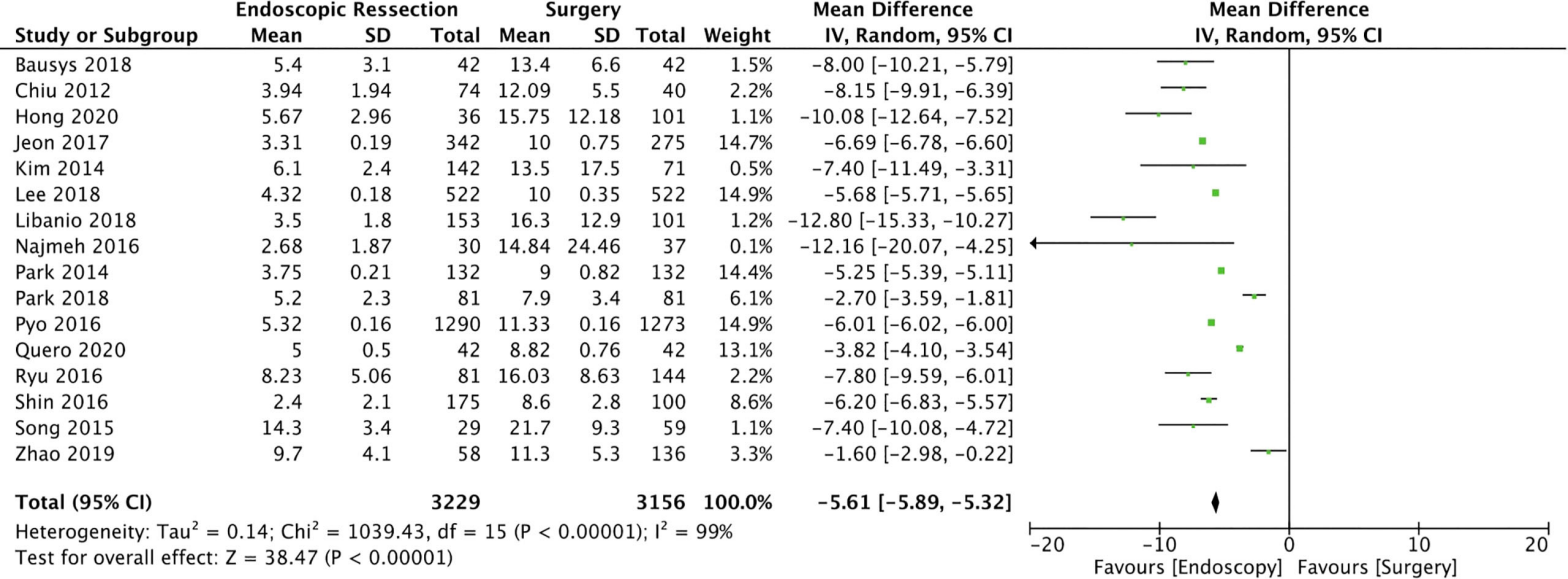
Forest Plot Length of Hospital Stay.

Sixteen studies (14, 16, 17, 20, 22, 23, 25, 26, 28, 31–35, 38, 40) were included in this analysis including 6385 patients (3229 in the EG group and 3156 in the SG group). The mean difference of hospital stay between ER and surgery was -5.61 days (95% CI -5.89, -5.32; I² = 99%; *p* < 0.00001), demonstrating a lower length of hospital stay for the ER group (**Figure 3**).

#### Adverse Events (AEs)

**Figure 4:**
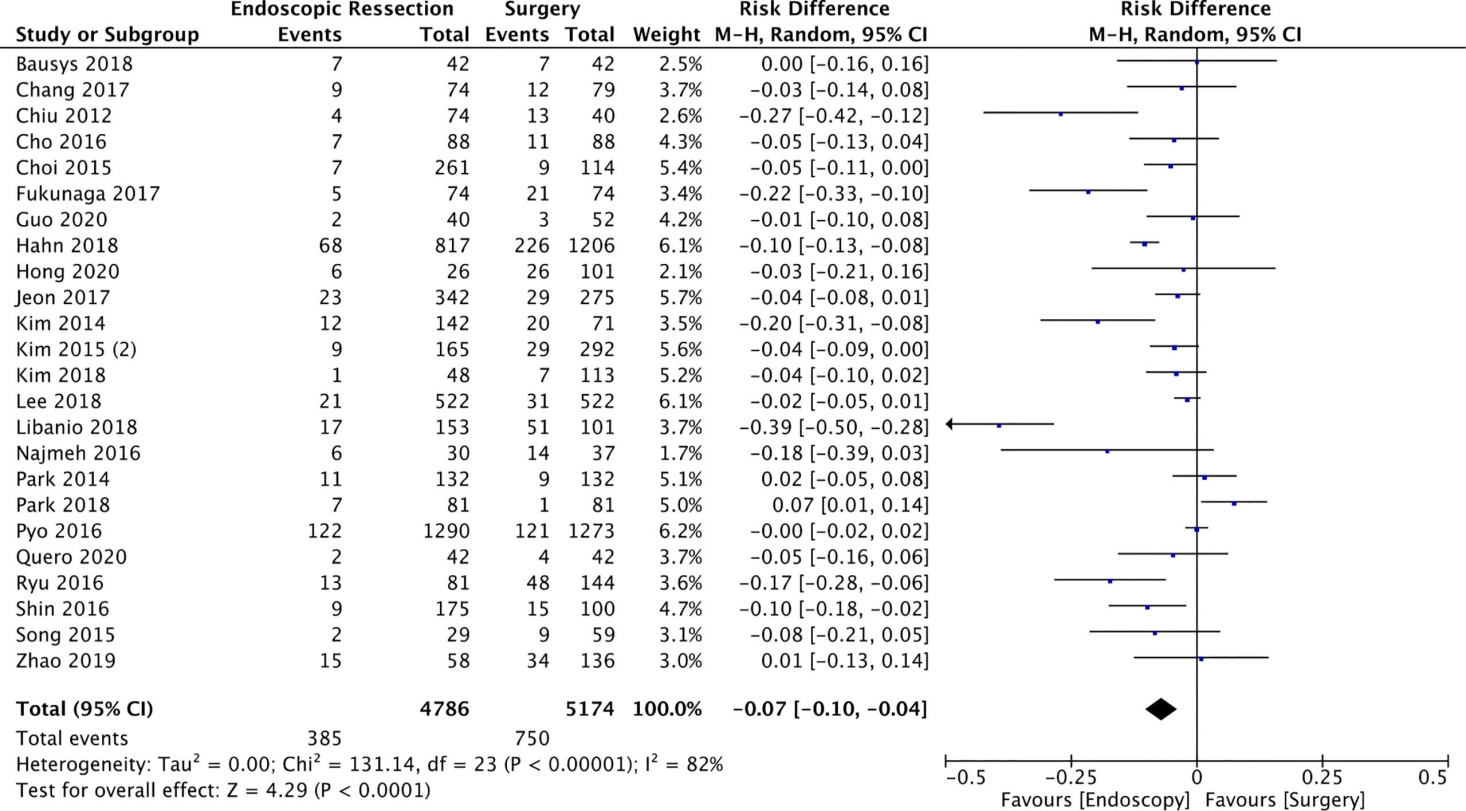
Forest Plot Adverse Events.

Adverse events were evaluated in 24 studies (14–23, 25–36, 38, 40) totaling 9960 patients (4786 in the ER and 5174 in the SG groups). The incidence of AEs was significantly lower in the ER group (RD = -0.07, 95%CI = -0.1, -0.04; I² = 82%; *p* < 0.0001), representing a number need to treat (NNT) of 14.28 (**Figure 4**).

#### Serious Adverse Events (AEs)

**Figure 5:**
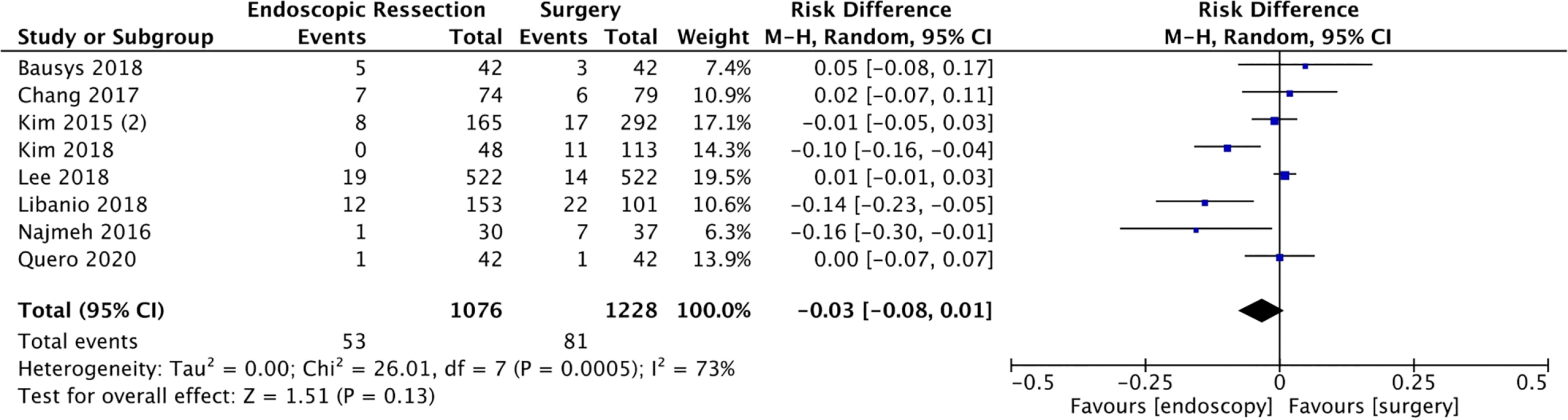
Forest Plot Serious Adverse Events.

Eight studies (14, 15, 17, 19, 23, 29, 35, 40) included in this analysis reported the severity of AEs, totaling 2304 patients (1076 in the ER and 1228 in the surgery groups). The pooled rates of serious AEs for ER and surgery were 4.9% and 6.6%, respectively, without statistically difference between both groups (RD = -0.03, 95%CI = -0.08, 0.01; I² = 73%; *p* = 0.13) (**Figure 5**).

#### Recurrence

**Figure 6:**
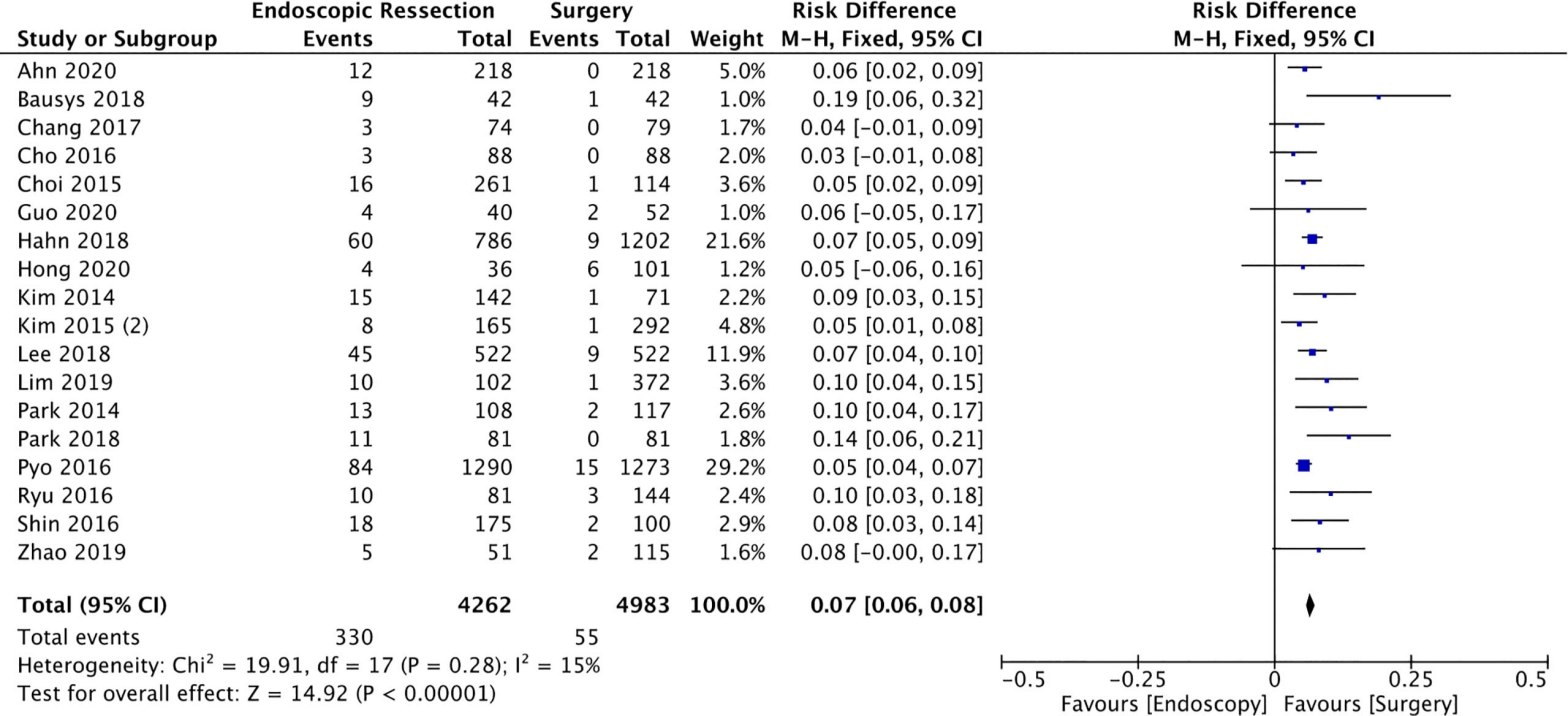
Forest Plot Recurrence.

Recurrence of gastric cancer was evaluated in 18 studies (13, 16–19, 22, 25–27, 29–32, 34, 36, 38–40) totaling 9245 patients (4262 in the ER and 4983 in the surgery group). The results showed lower incidence of recurrence in the group who underwent surgery (RD = 0.07, 95%CI = 0.06, 0.08; I² = 15%; *p* < 0.00001), representing a number need to harm (NNH) of 14.28 (**Figure 6**).

#### 5-Year Overall Survival

**Figure 7:**
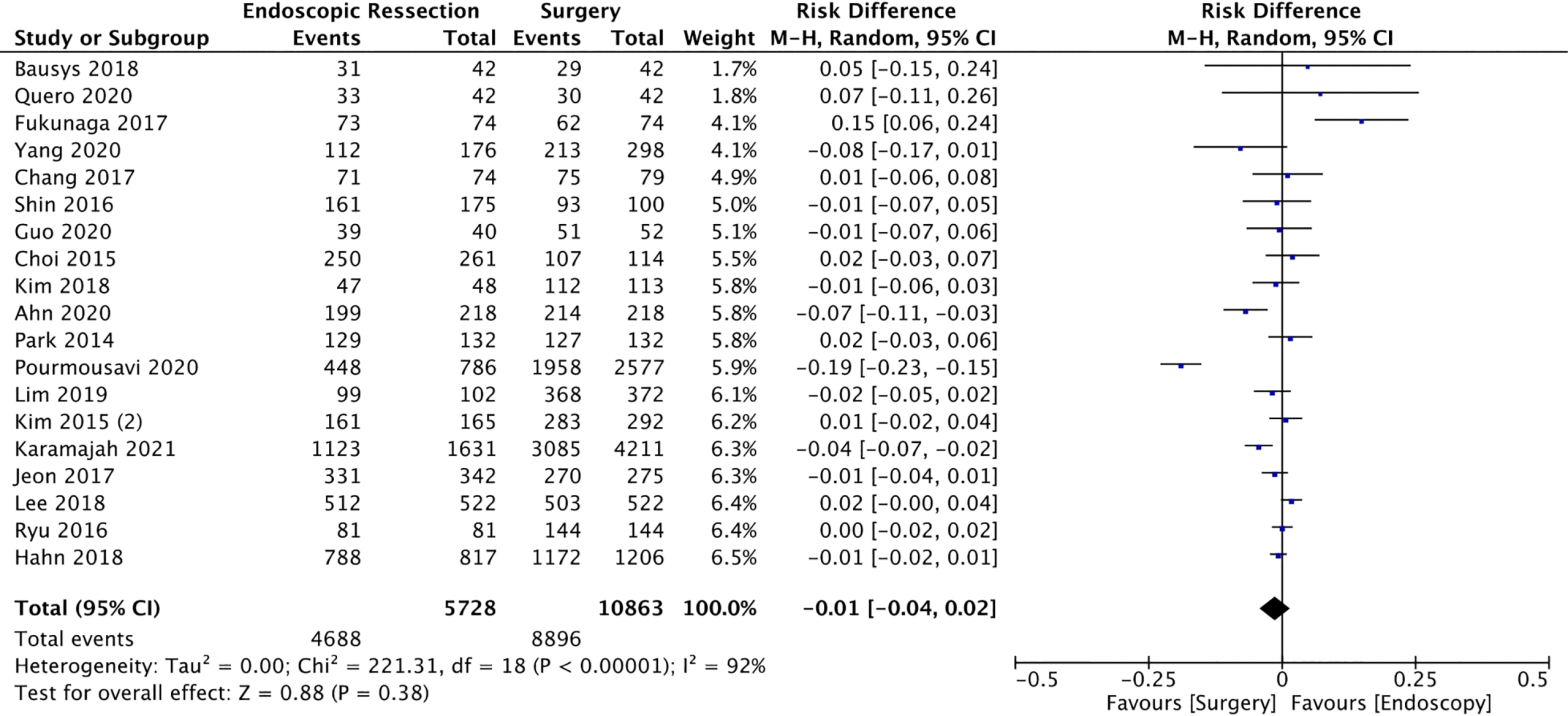
Forest Plot 5-year Overall Survival.

Five-year survival rate was evaluated in 19 studies (12, 13, 15, 17–22, 24, 25, 29–31, 35–37, 39, 40), including 16591 patients (5728 in the ER and 10863 in the surgery group). The overall survival was 81.8% in the ER and 81.9% in the surgery group. There was no statistical difference between groups (RD = -0.01, 95%CI = -0.04, 0.02; I² = 92%; *p* = 0.38) (**Figure 7**).

#### 5-Year Cancer-Specific Survival

**Figure 8:**
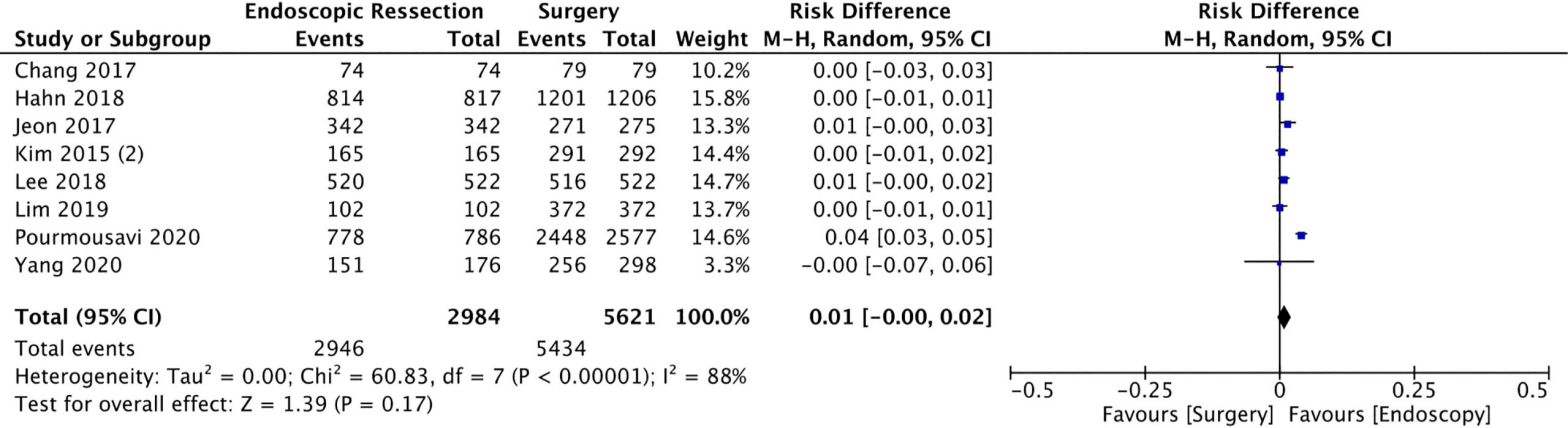
Forest Plot 5-year Cancer Specific Survival.

Eight studies (17–20, 24, 29, 37, 39) evaluated deaths caused only by gastric cancer to calculate cancer-specific survival, totaling 8605 patients (2946 in the EG and 5621 in SG). There was no difference of 5-year cancer-specific survival between the groups (RD = 0.01, 95%CI = 0.00, 0.02; I² = 88%; *p* < 0.17) (**Figure 8**).

## Discussion

Although gastrectomy is still considered the gold standard treatment for EGC, the endoscopic approach has emerged as an effective and less invasive therapy, both by EMR and ESD, especially for patients at low risk of lymph node metastasis, respecting Gotoda’s criteria (6, 7). There is no RCT comparing endoscopic versus surgical management of EGC, and previous meta-analyses did not incorporate the various relevant studies carried out over the last few years, which reflect the technical evolution of the endoscopic methods (41, 42).

To the best of our knowledge, this is the largest meta-analysis to date evaluating endoscopic versus surgical treatment for EGC (41, 42). Overall, the present study has several strengths. Firstly, several recently published studies, including a high number of patients were included, thus leading to more credible cumulative effects according to different outcome measures compared to previous meta-analyses (41, 42). Secondly, in addition to the analyses of survival and AEs, our study was able to compare the length of hospital stay and cancer-specific survival, providing a more accurate comparison of the effect of these interventions. Finally, GRADE methodology was used to assess the quality of all the included evidence.

According to the result of our study, a shorter length of hospital stay and lower rates of AEs were found in the ER when compared to the surgery group. Although our meta-analysis could not compare the cost difference between the modalities, both aforementioned outcomes favor lower hospital expenses related to hospitalization and operative complications, favoring a cheaper treatment, as seen in previous studies (43). A lower rate of serious AEs was expected for the ER group. However, there was no difference between serious AEs between both groups in this meta-analysis. This finding may be related to personal experience, as endoscopic resection techniques such as ESD, can be considered a novel approach when compared to surgery. Additionally, just a few studies have evaluated this outcome (8 of 29), and a low rate of serious AE were observed. Future studies evaluating this outcome may be necessary for a definitive conclusion.

As observed in previous studies, our meta-analysis reiterates the higher incomplete resection and incidence of cancer recurrence rates in patients undergoing ER. Several hypotheses may justify this finding. First, lower en-bloc resection rates and incomplete histological resection may correlate to higher recurrence in this group. Both the inexperience of the endoscopist and the narrower resection margin, when compared to surgery, can be related to the lower rate of complete resections in the group undergoing endoscopic treatment. Secondly, primary EGCs frequently develop in the middle or lower third of the stomach (44). The high-risk stomach portion is totally resected in a distal gastrectomy when the surgical approach is chosen. However, nearly the whole stomach is preserved after ESD, leading to a higher risk of recurrence in regions with intestinal metaplasia and glandular atrophy (45). Additionally, synchronous lesions may not be identified and removed in the ER group (42). Moreover, EGC patients, especially those meeting expanded ER criteria, carry a low risk of lymph node metastasis.

Although endoscopic treatment is associated with a higher recurrence and lower complete resection rates, as shown by our results, there was no difference in the overall 5-year mortality or cancer-specific mortality in this period. This is easily understood by the close follow-up that patients usually receive, enabling early re-diagnosis and rapid therapeutic reapproach, either by endoscopy or surgery. According to guidelines, surveillance endoscopies should be repeated every 6-12 months after ESD or EMR, even after curative resections. The interval can be shortened to 3-6 months in the first 3 years of follow-up (46).

Our systematic review and meta-analysis have some limitations. First, all 29 studies included studies are observational, leading to important drawbacks such as patient selection and information bias, amongst others. However, there is no RCT comparing endoscopy versus surgery in the treatment of EGC available in the literature, which makes these studies the best data available. Secondly, most of these studies (23 of 29) were conducted in Eastern countries (Japan, Korea, and China), where there is a higher prevalence of gastric cancer, specific screening programs, and greater expertise in performing advanced endoscopic resection procedures. On the other hand, many of the most recent (after 2015) studies included in this meta-analysis were carried out in the Western countries (6 of 23), reflecting the evolution and dissemination of endoscopic resection techniques, mainly ESD. Of the 7 outcomes evaluated in our study, 5 presented a low level of evidence according to the GRADE evaluation. The decrease in the level of evidence is mainly due to the nature of the studies, all observational, and the heterogeneity, which was greater than 50% in 6 and greater than 75% in 5 of the evaluated outcomes, being the use of random effect important to control the high heterogeneity. Additionally, since this is the largest meta-analysis on the subject and included studies published over the last 10 years, with patients undergoing the procedure since the early 2000s, it is understandable that with greater experience with the procedure there is a progressive change in its results. Moreover, the various centers that performed the procedures have their own protocols related to the procedure, care, and hospitalization, which could explain the high heterogeneity found.

In summary, due to similar 5-year mortality and cancer-specific mortality, endoscopic treatment is comparable to surgical treatment for the management of EGC. It is important to highlight that strict and careful monitoring should be applied to patients who received EMR or ESD for the treatment of EGC, given the association with higher recurrence and lower rates of complete resection. The patient must be aware of a planned follow-up program even before the procedure is performed. Additionally, given similar rates of serious AEs, with lower rates in total AEs and shorter length of hospital stay in the ER group, the endoscopic treatment appears to provide optimal maintenance of the quality of life and rapid recovery when compared to surgical management. Therefore, both procedures can be performed in patients with EGC, and the best approach should be individualized by considering personal and local experience, and the availability of material and devices.

## Conclusion

ER and surgery are safe and effective therapeutic approaches for ECG. ER provides lower rates of AEs and shorter hospital stays when compared to surgery. Despite that, ER is associated with lower complete resection rates and higher risk of recurrence, the 5-year mortality and cancer-specific mortality were similar between both approaches.

## Data Availability Statement

The original contributions presented in the study are included in the article/supplementary material. Further inquiries can be directed to the corresponding author.

## Author Contributions

AB: acquisition of data, analysis, interpretation of data, drafting the article, revising the article, and final approval; DM: analysis and interpretation of data, revising the article; IM: analysis and interpretation of data, revising the article; ES: acquisition of data, analysis, interpretation of data, drafting the article, revising the article, and final approval; IB: analysis and interpretation of data, revising the article; JR: analysis and interpretation of data, revising the article; analysis and interpretation of data, revising the article; SS-L: revising the article and english; WB: analysis and interpretation of data, drafting the article, final approval; EG: analysis and interpretation of data, drafting the article, revising the article, final approval. All authors contributed to the article and approved the submitted version.

## Conflict of Interest

The authors declare that the research was conducted in the absence of any commercial or financial relationships that could be construed as a potential conflict of interest.

The reviewer DS declared a shared affiliation with the authors AB, DM, IM, ES, IB, JR, AS, WB, and EG to the handling editor at the time of the review.

## Publisher’s Note

All claims expressed in this article are solely those of the authors and do not necessarily represent those of their affiliated organizations, or those of the publisher, the editors and the reviewers. Any product that may be evaluated in this article, or claim that may be made by its manufacturer, is not guaranteed or endorsed by the publisher.
